# Barriers and facilitators of adherence to awake prone positioning: a qualitative study using the COM-B model

**DOI:** 10.1186/s12890-023-02561-x

**Published:** 2023-07-19

**Authors:** Lingli Zhu, Zijun Ni, Yuping Zhang, Yang Zhan, Meijuan Lan, Ruiyi Zhao

**Affiliations:** 1 Nursing Department, The Second Affiliated Hospital of Zhejiang University School of Medicine, No.88 Jiefang road, Hangzhou, 310009 China; 2 Department of Nursing, School of Medicine, Zhejiang University, Hangzhou, China

**Keywords:** Awake prone position, COM-B model, Qualitative research

## Abstract

**Background:**

Awake prone positioning (APP) is a recommended therapy for non-intubated ARDS patients, but adherence can be challenging. Understanding the barriers and facilitators of adherence to APP is essential to increase the adherence of therapy and improve patient outcomes. The objective of this study was to explore the barriers and facilitators of adherence to awake prone ventilation using a qualitative approach and the Capability, Opportunity, Motivation-Behavior (COM-B) model.

**Methods:**

Semi-structured, in-depth interviews were conducted with patients involved in awake prone ventilation. Data were analyzed using an adapted inductive thematical approach and mapped onto the COM-B model to identify barriers and facilitators to adherence of APP.

**Results:**

Nineteen patients were interviewed (aged 55–92 years). Fifteen themes were identified and mapped directly on to the six COM-B constructs, with “physical challenges” related to physical capability being the primary barrier. These COM-B sub-items reflected five other barriers, including low self-efficacy(M), treatment environment(O), availability of time(O), misconceptions about the treatment(C), and insufficient knowledge(C). Key facilitators in adhering to APP were ability to identify and overcome obstacles(C), availability and affordability of treatment(O), family influences(O), beliefs and trust in treatment(M), fear about the disease(M), and perceived benefits(M). In addition, three factors played the role of both facilitator and barrier, such as media influences(O), healthcare influences(O), and behavioral habits(M).

**Conclusion:**

The COM-B model was proved to be a useful framework for identifying the barriers and facilitators of adherence to awake prone ventilation. The findings suggest that adherence behavior is a dynamic and balanced process and interventions aimed at improving adherence to APP should address the barriers related to capability, opportunity, and motivation. Healthcare providers should focus on providing proper guidance and training, creating a comfortable environment, and offering social support to improve patients’ capability and opportunity. Additionally, promoting patients’ positive beliefs and attitudes towards the treatment and addressing misconceptions and fears can further enhance patients’ motivation to adhere to the treatment plan.

## Introduction

Prone positioning is a well-established technique that has been demonstrated to improve oxygenation and reduce mortality in mechanically ventilated patients with moderate to severe acute respiratory distress syndrome (ARDS) [1]. This technique has also been applied to non-intubated ARDS patients, referred to as awake prone positioning (APP), with similar benefits [2–5]. APP means helping or encouraging an awake, spontaneously breathing patient to lie face down and move from a supine to a prone position [6]. During COVID-19 pandemic, healthcare providers have faced the growing need to reduce the demand for advanced respiratory care. The application of APP has shifted from patients undergoing vertebral body surgery, awake fiber-optic intubation, and lung transplantation [6–8] to non-intubated ARDS patients, which was supported by clinical guidelines and expert consensus statements [9–11]. The optimal duration of APP sessions remains uncertain, but previous studies indicated at least 8 h of daily prone position may be beneficial [2, 12]. A recent meta-analysis of over 4,000 COVID-19 patients revealed that the actual duration of APP varied significantly, ranging from 1–2 h to 8–10 h per day [13], which may potentially affect the effectiveness of treatment [13, 14]. A multicenter pragmatic randomized clinical trial showed that poor compliance was speculated as a possible factor for the lack of observed benefits [15]. It is thus necessary to identify the factors affecting patients’ difficulties in prolonged prone positioning and to create efficient therapies to increase adherence.

Limited research has been conducted to explore the patient experience with recommended treatment, and investigate the factors influencing the implementation of APP. Two recent qualitative studies have examined factors for implementing prone ventilation in mechanically ventilated patients from the perspective of healthcare professionals, including the attitudes and knowledge of healthcare workers, the characteristics of the ICU, as well as the influence of family, hospital, and social resources [16, 17]. Besides, it should be noted that unlike prone positioning with which patients are intubated, sedated, or even paralyzed, effective APP requires significant patient cooperation. Previous trials emphasized discomfort and fatigue as the most common barriers to patient adhesion [15, 18, 19], and patients’ knowledge, attitudes, and experience with APP impact adherence to prone ventilation therapy [19]. Even though quantitative studies have found factors that affect patient compliance, few interviews with non-intubated patients have been conducted to date, which can seek to produce a deeper, more complex, and all-encompassing understanding of experience, illness, or behavior [20].

**Fig. 1:**
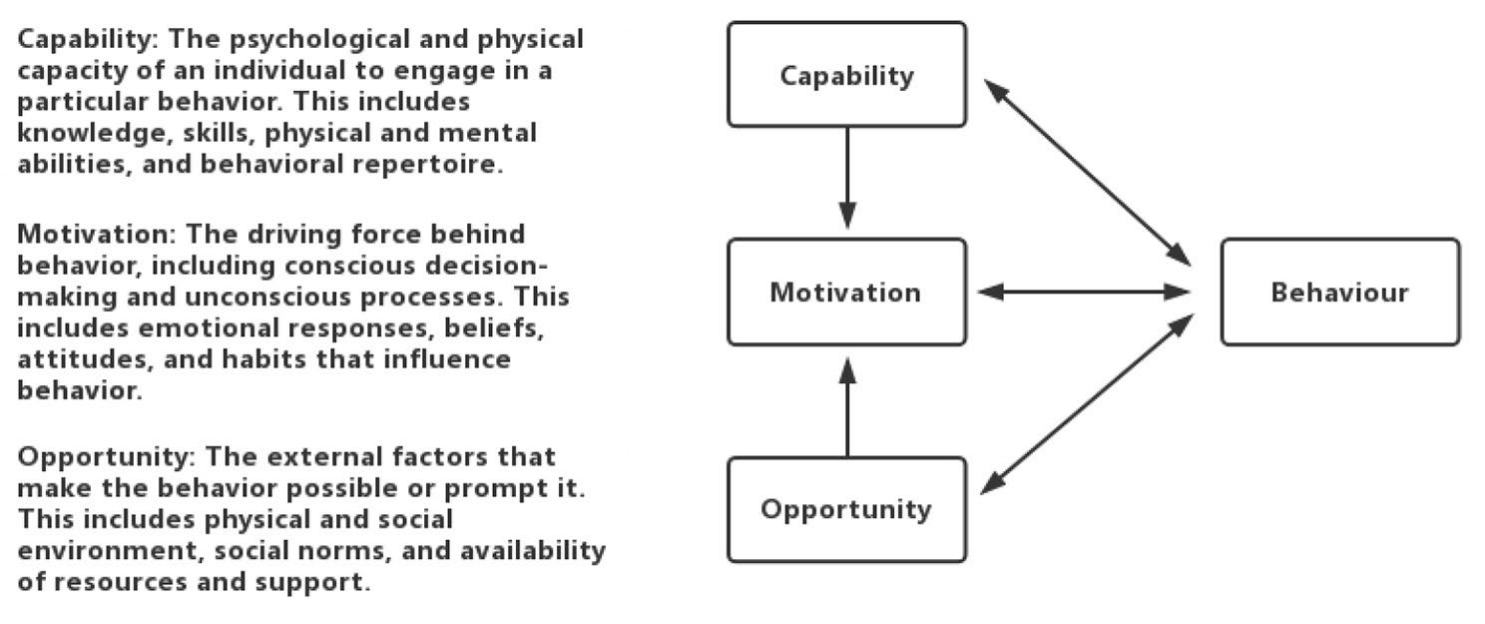
Capability, Opportunity, Motivation-Behavior (COM-B) model

Research suggests it can be most effective that using a theoretical framework to understand the behavior [21]. The Behavior Change Wheel (BCW) recommends using the Capability, Opportunity, Motivation-Behavior (COM-B) model (Fig. 1) to specify behavioral factors that can be targeted as part of behavior change intervention development [22]. This model highlights that the determinants of behavior include individuals’ physical and psychological abilities, physical and social opportunities, and reflective and automatic motivation [23]. Similar studies in other settings have been applied this model to understand behaviors, such as medication adherence [24] and physical activity [25] among patients. To gain insight into the patient experience with APP and gather in-depth feedback on factors that facilitate or deter its use, a qualitative study based on the COM-B model was conducted. The study aimed to identify factors that could be targeted to improve adherence to the treatment and enhance patient outcomes.

## Methods

### Study design

This is a descriptive qualitative study conducted in adherence to the Standards for Reporting Qualitative Research (SRQR) [26]. The data was collected using semi-structured qualitative interviews and analyzed through thematic analysis and COM-B mapping. The study obtained approval from the medical ethics committee of the Second Affiliated Hospital, Zhejiang University School of Medicine (No. I2023041).

### Participants

Participants in this study were recruited from a tertiary general hospital in Hangzhou, China, from January to February 2023. To ensure maximum variability, We used purposive sampling of patients with diverse characteristics (sex, age, education level, etc.), and we specifically selected patients with different duration of awake prone positioning for ventilation to capture a range of experiences. We excluded patients who refused or were unable to cooperate, or had altered mental status with inability to turn in bed without aid, and those who were treated in intensive care units or had communication disorders were also excluded. Sampling continued until thematic saturation was reached, and to confirm that no new information emerged, two additional participants were recruited for this study. Ultimately, 19 participants were included (See Table 1).

**Table 1:** Characteristics of participants

Characteristics	Categories	N(%)
**Age**	<60 ≥ 60	3 (15.8) 16(84.2)
	Mean ± SD	72.11 ± 12.95
**Sex**	Female	6 (31.6)
	Male	13(68.4)
**Education**	Primary and below Middle to high College and above	7 (36.8) 8 (42.1) 4 (21.1)
**Location**	Rural City	5(26.3) 14(73.7)
**Durations of prone positioning(h/d)**	≤ 3	11(57.9)
	4–10 >10 Mean ± SD	6 (31.6) 2 (10.5) 4.68 ± 3.67
**Durations of prone positioning(d)**	≤ 3 4–7 ≥ 7	11(57.9) 4 (21.1) 4 (21.1)
**Support/device**	O_2_ nasal cannula HFNC O_2_ mask NIV	10(52.6) 5(26.3) 3(15.8) 1(5.3)

HFNC: high-flow nasal cannula; NIV: noninvasive ventilation; O_2_:oxygen;

h/d: hours/days d: days

### Data collection and procedures

All potential participants were initially assessed and contacted by nurses, and informed them about the study. Once verbal consent was obtained, the first author scheduled appointments for the interviews. Prior to the interviews, written consent was obtained from each participant, with the assurance that they had the right to withdraw from participation at any time. The interviews started with the broad question “What is your experience of receiving awake prone ventilation?“ followed by specific items created based on the COM-B model to identify barriers and facilitators of patient adherence to awake prone positioning. Examples of such items include “Tell me a little bit about APP?“ and “What, for you, do you think are the benefits and risks of APP?“ The interviews were conducted in the wards, avoiding peak room visits and nursing care to ensure participant comfort. Digital audio-recorders and filed notes were used to record the interviews, which lasted between 28 and 40 min. To avoid researcher bias, the entire interview was completed by the same trained researcher.

### Data analysis

All interviews were transcribed verbatim by the first author within 24 h of the end of the interview, and then checked by the second author who listened to the recording to ensure accuracy. Any ambiguities were clarified with the interviewees in a timely manner to maximize the authenticity of the text. The transcribed data were organized and analyzed using NVivo12 software.

Using Braun & Clarke’s [27] analysis approach to inductive themes (Phase1) and map themes onto the COM-B model [23, 28] (Phase2). In phase 1, the first and second authors repeatedly read the transcribed texts to become familiar with the data and conducted primary coding to generate initial themes. Through further development and review, they refined, defined, and named the themes, and clarified the content. In phase 2, two authors reviewed the themes and representative quotes and mapped each theme onto the COM-B model. Any differences arising in both phases were discussed with the third author until a consensus was reached.

## Results

The study included participants with a mean age of 72 years, and 64.8% of them were male. The majority of participants(73.7%) resided in urban areas. Around 57.9% of the participants were ventilated in APP for less than 3 h per day, while only 10% received treatment for more than 10 h (See Table 1). Fifteen themes were extracted from the study that were found to have an impact of the treatment of APP in patients. These themes were closely related to the core elements of the COM-B model, as elaborated in the Table 2.

**Table 2:** Mapping of themes to the COM-B Model

Emerging sub-theme from the transcript	Sub-components of COM-B Model	Broad components of COM-B Model
1.Physical challenges (B)	Physical Capability	Capability
2. Insufficient knowledge(B) 3. Misconceptions about the treatment (B) 4. Ability to identify and overcome obstacles (F)	Psychological Capability	
5. Availability and affordability of treatment (F) 6. Availability of time (B) 7. Treatment environment (B)	Physical Opportunity	Opportunity
8. Family influences (F) 9. Media influences (M) 10.Healthcare influences (M)	Social Opportunity	
11. Beliefs and trust in treatment (F) 12. Fear about the disease(F) 13. Low self-efficacy (B)	Reflective Motivation	Motivation
14. Perceived benefits(F) 15. Behavior habits(M)	Automatic Motivation	

F: Facilitating factor B: Barrier factor M: Mix factor.

### Capabilities

#### Physical capability

There were many barriers and challenges to compliance with APP. Physical challenges was identified as a significant factor, particularly with regard to tolerance. Participants indicated that their bodies seemed unable to tolerate prolonged prone ventilation due to aging and their disease status, despite showing a positive attitude towards treatment. One participant mentioned, *“Prone position ventilation is a physical activity that requires a lot of energy…It’s not rest…it’s similar to exercising like Qigong for a long time.“(P3,Male).*

#### Psychological capability

Regarding psychological capability, participants in this study reported limited necessary knowledge and had many misconceptions about the treatment. During the course of treatment, individuals frequently adopted inappropriate prone positions. As one participant described: *“I’m not sure…I just lay on me bed, even though it really makes me uncomfortable.”(P14,Female).* Additionally, some expressed concerns about the potential harm and lack of information provided by healthcare professionals. For example, one participant stated: *“I just don’t know what it does for my body…you know what? That’s against my common sense, in my view, keeping the prone position will damage my heart…”(P14, Female).* Another participant believed that staying in the position for too long could lead to suffocation and expressed fears about it: *“I am worried about taking an APP for a long time, no one told me if something was going to happen…”(P18,Male).* Moreover, participants had many misconceptions about the treatment of APP, especially regarding its details. As one participant said, *“When my oxygen saturation can stay above 90%, I thought it was time to stop. Actually, I am only keeping it up for a few minutes…”(P2, Female).* However, some participants demonstrated a greater capacity for problem-solving and were able to overcome obstacles. They made adjustments to their position, improved their equipment, or sought assistance to prolong the duration of ventilation. For example, one participant found a solution to the discomfort caused by the prone position by using a pillow with a hollow hole in the middle to avoid neck strain and pressure on their arms. He explained, *“I realized that I could maintain this position for a long time when I went for a massage…I asked my daughter to buy a pillow with a hollow hole in the middle (similar to a swimming ring or a donut)…In fact, it worked very well…”(P6, Male).*

### Opportunity

#### Physical opportunity

The physical opportunity was identified as a crucial facilitator of the awake prone ventilation treatment. The availability and affordability of the treatment were emphasized as important factors that positively influenced patient behavior. Participants noted that the simplicity of the execution and the low cost of the treatment made it a viable option compared to other treatments such as oxygen inhalation or mechanical ventilation. One participant articulated, *“Just a simple prone position can make you feel better…why not do that?” (P13, Male).* Further, the awake prone position places greater emphasis on active patient cooperation than the prone position for intubated sedated patients. However, participants noted that the availability of time was a challenge. For instance, one participant noted, *“With an IV injection one minute and a bathroom break the next… I don’t have time to perform prone ventilation, and I think I’ll have full time to do it (APP) when I get home…” (P15, Male).*

Moreover, participants identified the treatment environment as a key factor that can influence patient behavior. Many patients highlighted that the presence of therapeutic tubes or devices could hinder their ability to change or adapt their position actively. For example, one participant explained, *“I have a catheter in my neck (CVC)…I can’t adjust my posture at will…” (P11, Male).* Similarly, another participant expressed, *“Actually, you can see that I had so many tubes in my body that I couldn’t move around like a normal person, especially in the prone position…”(P18, Male).*

#### Social opportunities

Social opportunities emerged as an important theme in this study, as participants described how their caregiver attitudes, caregiver support, and family relationships influenced their treatment adherence behaviors. One participant stated, *“I couldn’t turn over on my own effectively…my daughter told me it (APP) was good for my health, so she assists me with these treatments during the day. She encourages and supervises me to complete these tasks every day, even if sometimes I try to be lazy…” (P9, Male).* This highlights that for some participants, supportive family members prompts adherence in APP. Another important aspect of social opportunities highlighted by participants was the use of short-form video and internet platforms such as We Chat for medical science popularization in China. Several participants reported that they found all the available online information helpful in understanding and accepting new medical measures. For instance, one participant said, *“I read all the information on your hospital’s app, and I know that APP is good…”(P11, Male)*. However, some participants also expressed concerns about the abundance of online information and the challenge of distinguishing between useful and practical methods, as one participant noted, *“There is too much information online, and it’s hard to tell what’s really practical.”(P8, Male).* Participants also described how the attention given to treatment by healthcare providers could improve their compliance behavior. For example, one participant stated, *“The doctors and nurses remind me several times a day… At first, I was afraid of the prone position, but after receiving communication and guidance from the nurses, I gradually accepted it…” (P15, Male).* This highlights the importance of healthcare providers in providing social opportunities for patients to adhere to treatment plans. However, some participants also expressed concerns about inadequate communication with healthcare providers. Some felt that they did not receive enough information about the treatment from their doctors, which hindered their ability to comply with it. For instance, one participant stated, *“I’m afraid the catheter will come out… they don’t explain in detail… They always say ‘It’s good for your lung function…’ but I need to know what risks I might be exposed to…”(P12, Female).* Another participant added, *“They looked so busy that I didn’t dare to disturb them too much.” (P14, Female).*

### Motivation

#### Reflexive motivation

Reflexive motivation, which involves beliefs, trust, emotions, and reflective processes, played a crucial role in behavior modification among participants in this study. Participants had faith in the treatment, especially in the visual effects of APP, which motivated them to adhere to the recommended behavior. Some participants reported significant improvements in their oxygen saturation and sputum expectoration after practicing APP, which encouraged them to continue with the treatment. For example, one participant stated, *“While practicing, I would watch the monitor, and to my surprise, my oxygen saturation increased significantly to 96%… I was just doing something as simple as that…” (P11, Male).* Fear of the consequences of the disease also acted as a motivator for behavior modification. Participants were scared that if they did not cooperate well with the treatments, their condition might worsen to the point where they needed to be put on ventilators. As one participant expressed, *“I want to get well quickly… I don’t have a choice. The doctor said if I don’t want to get worse, I need to practice it…”(P9, Male).* However, participants also expressed doubts about their ability to adhere to APP, indicating low self-efficacy. They felt that practicing daily and attending the required sessions would be too much effort. As one participant expressed, *“And then I don’t have any confidence to promise that I will keep going…” (P19, Male).*

#### Automatic motivation

In this study, two factors that influence automatic motivation are the behavior habits and perceived benefits. Participants who had already formed the habit of using the prone position were more likely to be motivated to continue using it. For example, one participant reported that lying on their stomach became easier over time and reduced feelings of tiredness (P9, Male). In addition, perceived benefits, such as improved breathing and overall health, also contribute to automatic motivation. One participant said, *“You know, it’s important to have taken care of it before the problem, I can practice it (APP) to avoid endotracheal intubation…”(P6, Male).* However, some participants reported experiencing difficulties in changing their habits, which presents a significant barrier to their motivation. One participant aptly described, *“It’s uncomfortable for me to lie on my stomach and I could not tolerate the sensation… I am used to sleeping on my back.” (P4, Male).*

## Discussion

In this study, we identified 15 themes that completely matched the COM-B model, providing a comprehensive understanding of the factors that affect patient adherence behavior [23]. Our findings suggest that adherence to the prone position among patients is a dynamic balance process that is influenced by capability, motivation, and opportunity.

Unlike a study in Mexico, the mean duration of prone position in our study was only 4.68 h per day, significantly lower than its 8.6 h [29]. Physical weakness appeared to be a significant barrier, particularly as our patients were older and had greater difficulty overcoming the discomfort caused by stiffness and pain [18, 30]. This obstacle may be addressed through strategies such as using additional pillows or rolled towels [18], and the adoption of novel positions like the dolphin prone position [31], Reverse Trendelenburg position [11], alternating prone positioning [32], and Rodin’s position [33]. Our study also found that the lack of knowledge and misconceptions about prone position were major factors affecting patients’ psychological capability, which may be related to their limited ability to understand and accept information, similar to the survey by Sethi et al [19]. Moreover, these factors may also lead to patients’ fear of prone position ventilation, thereby weakening their confidence in adhering to the prescribed course of action. To address these barriers, various educational methods can be used, such as providing detailed information by handouts to reduce patients’ insecurity during treatment [34], as emphasized by the Behavior Change Wheel [23].

When it comes to physical opportunities, it also illustrates that treatment accessibility is another crucial factor affecting patient compliance, particularly for sober patients [15], and that the impact of the treatment environment should not be overlooked [35]. In order to improve patient compliance, it is necessary to consider the physical and psychological needs of the patient and to choose an appropriate time for treatment (e.g., at night in the prone position) to minimize environmental disturbances. Optimizing treatment environments, such as placing electrodes on the back, substituting nasal catheters for masks for oxygen inhalation, and providing hollow pillows to relieve discomfort can address patients’ physical needs, which is a component of the COM-B model. Social opportunity is also a key factor in promoting patient compliance. In the ward, caregivers play a crucial role in assisting with patient care and supervising the implementation of treatment. Empowering education for family members can not only help patients better adhere to APP but also reflect the importance of humanized care. At the same time, releasing information about treatment at the hospital level through Internet, patients’ acceptance of treatment can be improved, and the shortage of clinical nursing resources in the ward also can be alleviated.

Based on the COM-B model, the factors related to capability and opportunity may further improve or inhibit patients’ motivation to adhere to the APP [23]. As described in this study, poor self-efficacy due to a lack of capability hindered patient evaluation of the course of treatment and impeded adherence, which is consistent with previous researches [36, 37]. Additionally, the study found a new result that daily habits had both positive and negative effects on adherence. Those who prefer to sleep on their stomach can last longer in treatment, but for others, it can be a difficult posture to maintain and an uneasy maneuver to perform [19]. Notably, participants with higher compliance reported an increased tolerance for the prone position over time, suggesting that healthcare professionals could encourage patients to gradually increase the duration of each session and try the prone position several times throughout the day. Furthermore, the current study also identified fear of disease, perceived benefits, and a strong belief in treatment as facilitators of initiation and maintenance of APP, with a positive feedback mechanism between these factors. Participants stated that their fear of disease severity and potential complications was the driving force behind their decision to initiate and comply with treatment. As they began to experience positive changes from the treatment, their trust in the treatment approach increased, further solidifying their adherence. Interestingly, the study found that patients’ motivational change was a process of calculated risk, similar to findings in sexual health behavior research where risk-benefit analyses affect motivation [38]. These findings indicate that these obstacles can be considered alongside education around the health consequences in order to motivate adherence to prone position therapy to begin with. Further interventions can be made to reinforce positive beliefs through education and clear communication from healthcare professionals about the benefits of the prone position.

### Strengths and limitations

This study adds to the existing limited literature and explores the perceptions of awake patients regarding prone ventilation therapy. A noteworthy strength lies in the theoretical analysis of the factors influencing compliance behavior with prone ventilation therapy in awake patients. Utilization of the COM-B model allows the results of this study to be fed directly into the behavioral analysis to facilitate the development of empirically and theoretically based behavior change interventions, which is the first stage of intervention development in line with BCW theory [23]. As well, a heterogeneous sample of patients undergoing awake prone ventilation was recruited in terms of social characteristics (age, gender, literacy) and duration of prone ventilation, which allowed for a wider range of perspectives to be included in the analysis.

Notwithstanding, there were several limitations. Firstly, this study was conducted in a specific healthcare setting with a small sample size, which may limit the generalizability of the findings to other contexts. Secondly, the study relied on self-reported data from patients, which may be subject to recall bias and social desirability bias. Additionally, the study did not include perspectives from healthcare providers, which could provide valuable insights into factors that influence adherence to awake prone ventilation. Despite these limitations, this study provides a valuable contribution to the literature on adherence to awake prone ventilation and highlights the importance of considering behavioral determinants when designing interventions to improve patient adherence to this therapy.

### Implications and future direction

The results of this study were found based on the COM-B model from the patient’s perspective, but further research to extend the findings is needed. Future studies could incorporate multiple healthcare settings to delve into how “opportunity” may impact adherence to APP, and utilize mixed research methods and triangulation of data from different sources [39], such as patient self-reports and health provider perspectives, to gain a more comprehensive understanding of the factors influencing patient behavior.

The findings of the current study can be used as a basis for developing interventions to promote patient adherence to APP. All the factors identified in the present study are interrelated and a simple suggestion of “self-proning” is not enough as a therapeutic intervention to achieve prolonged adherence to APP [13, 40, 41]. Further clinical practice and intervention research can develop targeted measures that address specific barriers to adherence by effectively utilizing the intervention functions within the BCW theory [23]. For example, providing adequate education (increasing knowledge or understanding) and using persuasion (persuasive communication to induce positive or negative feelings) can be effective strategies for reducing barriers. Additionally, the integration of digital technologies in the implementation of APP could be beneficial in addressing physical challenges and the scarcity of human resources. For instance, virtual reality, a digital technology for achieving personalized exercise and participation motivation, has shown promise in the field of respiratory rehabilitation [42, 43]. Future studies could further explore the potential of digital technologies and determine their optimal use in APP clinical settings.

## Conclusion

In conclusion, this study provides valuable insights into the barriers and facilitators of adherence to awake prone ventilation using the COM-B model, which is influenced by the interplay between capability, opportunity, and motivation. Physical capability, particularly weakness, is the main factor that impedes patients from maintaining prone position ventilation for a prolonged period. However, opportunities for support, such as equipment and family support, and psychological adaptation can alleviate the negative impact of physical limitations. Moreover, patients’ motivation—including beliefs about treatment and fear of disease complications—can influence their adherence to prone position ventilation positively if proper communication is maintained to deliver accurate treatment-related information. Overall, the study’s findings have important implications for healthcare professionals and policymakers in designing tailored interventions that target specific adherence barriers, enhance patient education, and improve the quality of healthcare services. Future research should investigate the effectiveness of implementing strategies based on the COM-B model to enhance patients’ adherence behavior.

## Data Availability

The datasets used and/or analyzed during the current study are available from the corresponding author on reasonable request.

## Abbreviations

APP: Awake prone positioning
ARDS: Acute respiratory distress syndrome
BCW: Behavior Change Wheel
COM-B: Capability, Opportunity, Motivation-Behavior
CVC: Central venous catheter

## References

[1] Scaravilli V, Grasselli G, Castagna L, Zanella A, Isgrò S, Lucchini A (2015). Prone positioning improves oxygenation in spontaneously breathing nonintubated patients with hypoxemic acute respiratory failure: a retrospective study. J Crit Care.

[2] Rampon GL, Simpson SQ, Agrawal R (2023). Prone positioning for Acute Hypoxemic respiratory failure and ARDS: a review. Chest.

[3] Ding L, Wang L, Ma W, He H (2020). Efficacy and safety of early prone positioning combined with HFNC or NIV in moderate to severe ARDS: a multi-center prospective cohort study. Crit Care.

[4] Massart N, Guervilly C, Mansour A, Porto A, Flécher E, Esvan M (2023). Impact of prone position in COVID-19 patients on extracorporeal membrane oxygenation. Crit Care Med.

[5] Okin D, Huang C-Y, Alba GA, Jesudasen SJ, Dandawate NA, Gavralidis A (2023). Prolonged prone position ventilation is Associated with reduced mortality in intubated COVID-19 patients. Chest.

[6] Fusi C, Bulleri E, Villa M, Pisani L, El Aoufy K, Lucchini A (2023). Awake Prone Positioning in Nonintubated patients with Acute Hypoxemic Respiratory failure. Crit Care Nurse.

[7] Malcharek MJ, Rogos B, Watzlawek S, Sorge O, Sablotzki A, Gille J (2012). Awake fiberoptic intubation and self-positioning in patients at risk of secondary cervical injury: a pilot study. J Neurosurg Anesthesiol.

[8] Mahrous RSS, Ahmed AMM (2018). The Shikani Optical Stylet as an alternative to Awake Fiberoptic Intubation in patients at risk of secondary cervical spine Injury: a Randomized Controlled Trial. J Neurosurg Anesthesiol.

[9] Notice on the issuance of the treatment protocol for novel coronavirus infection. (trialversion10). http://www.nhc.gov.cn/ylyjs/pqt/202301/32de5b2ff9bf4eaa88e75bdf7223a65a.shtml. Accessed 30 Mar 2023.

[10] Oxygenation and Ventilation for Adults. COVID-19 Treatment Guidelines. https://www.covid19treatmentguidelines.nih.gov/management/critical-care-for-adults/oxygenation-and-ventilation-for-adults/. Accessed 30 Mar 2023.

[11] ICS Guidance for Prone Positioning of the Conscious COVID Patient. 2020. ICM Anaesthesia COVID-19.https://icmanaesthesiacovid-19.org/news/ics-guidance-for-prone-positioning-of-the-conscious-covid-patient-2020. Accessed 30 Mar 2023.

[12] Li J, Roca O, Ehrmann S (2023). Prone positioning of nonintubated patients with acute hypoxemic respiratory failure. Curr Opin Crit Care.

[13] Li J, Luo J, Pavlov I, Perez Y, Tan W, Roca O (2022). Awake prone positioning for non-intubated patients with COVID-19-related acute hypoxaemic respiratory failure: a systematic review and meta-analysis. Lancet Respir Med.

[14] Chen L, Zhang Y, Li Y, Song C, Lin F, Pan P (2022). The application of Awake-Prone Positioning among non-intubated patients with COVID-19-Related ARDS: a narrative review. Front Med (Lausanne).

[15] Fralick M, Colacci M, Munshi L, Venus K, Fidler L, Hussein H (2022). Prone positioning of patients with moderate hypoxaemia due to covid-19: multicentre pragmatic randomised trial (COVID-PRONE). BMJ.

[16] Klaiman T, Silvestri JA, Srinivasan T, Szymanski S, Tran T, Oredeko F (2021). Improving prone positioning for severe Acute Respiratory Distress Syndrome during the COVID-19 pandemic. An implementation-mapping Approach. Ann Am Thorac Soc.

[17] Hochberg CH, Card ME, Seth B, Kerlin MP, Hager DN, Eakin MN (2023). Factors influencing the implementation of Prone Positioning during the COVID-19 pandemic: a qualitative study. Ann Am Thorac Soc.

[18] Jha A, Chen F, Mann S, Shah R, Abu-Youssef R, Pavey H (2022). Physiological effects and subjective tolerability of prone positioning in COVID-19 and healthy hypoxic challenge. ERJ Open Res.

[19] Sethi SM, Hirani S, Iqbal R, Ahmed AS (2022). Patient’s perspective of Awake Proning: a cross-sectional interview-based Survey from COVID-19-Recovered patients. Crit Care Explor.

[20] Ee C, MacMillan F, Boyages J, McBride K (2022). Barriers and enablers of weight management after breast cancer: a thematic analysis of free text survey responses using the COM-B model. BMC Public Health.

[21] Glanz K, Bishop DB (2010). The role of behavioral science theory in development and implementation of public health interventions. Annu Rev Public Health.

[22] The Behaviour Change Wheel Book - A Guide. To Designing Interventions. https://www.behaviourchangewheel.com/. Accessed 30 Mar 2023.

[23] Michie S, van Stralen MM, West R (2011). The behaviour change wheel: a new method for characterising and designing behaviour change interventions. Implement Sci.

[24] Heneghan MB, Hussain T, Barrera L, Cai SW, Haugen M, Duff A (2020). Applying the COM-B model to patient-reported barriers to medication adherence in pediatric acute lymphoblastic leukemia. Pediatr Blood Cancer.

[25] Flannery C, McHugh S, Anaba AE, Clifford E, O’Riordan M, Kenny LC (2018). Enablers and barriers to physical activity in overweight and obese pregnant women: an analysis informed by the theoretical domains framework and COM-B model. BMC Pregnancy Childbirth.

[26] O’Brien BC, Harris IB, Beckman TJ, Reed DA, Cook DA (2014). Standards for reporting qualitative research: a synthesis of recommendations. Acad Med.

[27] Braun V, Clarke V (2006). Using thematic analysis in psychology. Qualitative Res Psychol.

[28] Berry E, Jenkins C, Allen S (2022). Using the COM-B model to characterize the barriers and facilitators of pre‐exposure prophylaxis (PrEP) uptake in men who have sex with men. BMC Public Health.

[29] Ehrmann S, Li J, Ibarra-Estrada M, Perez Y, Pavlov I, McNicholas B (2021). Awake prone positioning for COVID-19 acute hypoxaemic respiratory failure: a randomised, controlled, multinational, open-label meta-trial. Lancet Respir Med.

[30] Shikano K, Sakao S, Inaba Y, Taniguchi T, Saito G, Naito A (2022). Tolerability of prone positioning in non-intubated patients with hypoxaemia due to COVID-19-related pneumonia. Respirology.

[31] Lucchini A, Minotti D, Vanini S, Pegoraro F, Iannuzzi L, Isgrò S (2021). The “Dolphin” prone position in Awake COVID-19 patients. Dimens Crit Care Nurs.

[32] Bentley SK, Iavicoli L, Cherkas D, Lane R, Wang E, Atienza M (2020). Guidance and patient instructions for Proning and Repositioning of Awake, Nonintubated COVID-19 patients. Acad Emerg Med.

[33] Coppo A, Winterton D, Benini A, Monzani A, Aletti G, Cadore B (2021). Rodin’s thinker: an alternative position in Awake patients with COVID-19. Am J Respir Crit Care Med.

[34] Paul V, Patel S, Royse M, Odish M, Malhotra A, Koenig S (2020). Proning in Non-Intubated (PINI) in Times of COVID-19: Case Series and a review. J Intensive Care Med.

[35] Bhandari B, Narasimhan P, Vaidya A, Subedi M, Jayasuriya R (2021). Barriers and facilitators for treatment and control of high blood pressure among hypertensive patients in Kathmandu, Nepal: a qualitative study informed by COM-B model of behavior change. BMC Public Health.

[36] Ha FJ, Hare DL, Cameron JD, Toukhsati SR (2018). Heart failure and Exercise: a narrative review of the role of self-efficacy. Heart Lung Circ.

[37] Hu Y, Chen X, Fan J, Huang Y, Ye J, Gu F (2023). The subjective Will and Psychological Experience of Home-Based Exercise in Lung Cancer Patients during interval of chemotherapy: a qualitative study. J Multidiscip Healthc.

[38] Madhani A, Finlay KA (2022). Using the COM-B model to characterize the barriers and facilitators of pre‐exposure prophylaxis (PrEP) uptake in men who have sex with men. Br J Health Psychol.

[39] Carter N, Bryant-Lukosius D, DiCenso A, Blythe J, Neville AJ (2014). The use of triangulation in qualitative research. Oncol Nurs Forum.

[40] McNicholas BA, Ibarra-Estrada M, Perez Y, Li J, Pavlov I, Kharat A (2023). Awake prone positioning in acute hypoxaemic respiratory failure. Eur Respir Rev.

[41] Rampon G, Jia S, Agrawal R, Arnold N, Martín-Quirόs A, Fischer EA (2022). Smartphone-guided self-prone positioning vs Usual Care in Nonintubated Hospital Ward patients with COVID-19. Chest.

[42] Colombo V, Aliverti A, Sacco M (2022). Virtual reality for COPD rehabilitation: a technological perspective. Pulmonology.

[43] Rutkowski S, Bogacz K, Rutkowska A, Szczegielniak J, Casaburi R (2023). Inpatient post-COVID-19 rehabilitation program featuring virtual reality-preliminary results of randomized controlled trial. Front Public Health.

